# Comparison of the Accuracy of Voxel Based Registration and Surface Based Registration for 3D Assessment of Surgical Change following Orthognathic Surgery

**DOI:** 10.1371/journal.pone.0093402

**Published:** 2014-04-02

**Authors:** Anas Almukhtar, Xiangyang Ju, Balvinder Khambay, James McDonald, Ashraf Ayoub

**Affiliations:** 1 Orthodontic Department, Glasgow Dental School, University of Glasgow, Glasgow, United Kingdom; 2 Medical Device Unit, Department of Clinical Physics and Bioengineering, NHS Greater Glasgow and Clyde, Glasgow, United Kingdom; 3 Honorary Research Fellow, Glasgow Dental School, University of Glasgow, Glasgow, United Kingdom; 4 Discipline of Orthodontics and Paediatric Dentistry, Faculty of Dentistry, The University of Hong Kong, Hong Kong, Hong Kong SAR; 5 Oral and Maxillofacial Surgery Department, Glasgow Dental School, University of Glasgow, Glasgow, United Kingdom; Institute of Psychology, Chinese Academy of Sciences, China

## Abstract

**Purpose:**

Superimposition of two dimensional preoperative and postoperative facial images, including radiographs and photographs, are used to evaluate the surgical changes after orthognathic surgery. Recently, three dimensional (3D) imaging has been introduced allowing more accurate analysis of surgical changes. Surface based registration and voxel based registration are commonly used methods for 3D superimposition. The aim of this study was to evaluate and compare the accuracy of the two methods.

**Materials and methods:**

Pre-operative and 6 months post-operative cone beam CT scan (CBCT) images of 31 patients were randomly selected from the orthognathic patient database at the Dental Hospital and School, University of Glasgow, UK. Voxel based registration was performed on the DICOM images (Digital Imaging Communication in Medicine) using Maxilim software (Medicim-Medical Image Computing, Belgium). Surface based registration was performed on the soft and hard tissue 3D models using VRMesh (VirtualGrid, Bellevue City, WA). The accuracy of the superimposition was evaluated by measuring the mean value of the absolute distance between the two 3D image surfaces. The results were statistically analysed using a paired Student *t*-test, ANOVA with post-hoc Duncan test, a one sample t-test and Pearson correlation coefficient test.

**Results:**

The results showed no significant statistical difference between the two superimposition methods (p<0.05). However surface based registration showed a high variability in the mean distances between the corresponding surfaces compared to voxel based registration, especially for soft tissue. Within each method there was a significant difference between superimposition of the soft and hard tissue models.

**Conclusions:**

There were no significant statistical differences between the two registration methods and it was unlikely to have any clinical significance. Voxel based registration was associated with less variability. Registering on the soft tissue in isolation from the hard tissue may not be a true reflection of the surgical change.

## Introduction

Traditionally skeletal and soft tissue changes following orthognathic surgery have been assessed in two dimensions by superimposing pre- and post-operative lateral cephalographs on stable skeletal structures such as the anterior cranial base [1], [2] or by comparing linear and angular cephalometric measurements [3].

The use of low dose cone beam CT scans (CBCT) has now allowed capture of the skeletal and soft tissues in three-dimensions [4], [5]. Quantifying the surgical changes using 3D images follows the same method as the traditional 2D analyses with the addition of the third dimension (the depth), which augments the amount of information obtained from the facial image [6], [7]. However, the superimposition technique of pre- and post-operative 3D images is more complex due to the 3D nature of the images. The output image volume is composed of small units called voxels, the dimensions of which depend on the selected image resolution. Voxels are volumetric units with isotropic x, y, and z dimensions stored in a DICOM format (Digital Imaging Communication in Medicine). Each voxel has a unique grey scale value which depends on the opacity of the structure scanned in that volume [8].

The 3D volumetric image can be converted into 3D surface models using mathematical algorithms such as the Marching cubes algorithm [9]. The 3D rendered model can then be used for visualising the skeletal or soft tissue anatomical surfaces.

Surface based registration (SBR) was the initial method described for 3D image superimposition [10], [11]. The principle involves approximating two surfaces by selecting corresponding landmarks on the two images and translating and rotating one of the images so the landmarks align. This is followed by an iterative process (Iterative Closest Point (ICP) algorithm) which minimises the surface distance between the two surfaces. This type of registration is often referred to as surface based registration. Recently, a new method was introduced to the medical research field known as voxel based registration (VBR). It has been widely used for various medical applications and research purposes, including diagnoses, treatment planning and assessment of a variety of cases utilizing CT, CBCT, MRI, and 3D ultrasound [12]–[15].

Voxel based registration utilizes the grey scale difference of the voxels to align the two DICOM images to the best superimposition achieving the least total grey scale density difference between the two images. Voxel-based registration uses the intensities throughout the entire selected volume and therefore uses the image content as the basis of the registration and is useful were it is difficult to detect distinct surface topography features.

Studies reporting the use of voxel based registration have claimed high accuracy in registration [16]–[18]. However, to date, no research has been published comparing the accuracy of voxel based and surface based registration methods.

### Aim

The objective of this study was to determine if there was a statistically significant difference in the accuracy of image superimposition between two registration methods i.e. surface based and voxel based. This comparative assessment between the two methods of superimposition has not been previously reported.

## Materials and Methods

### Data Capture

This is a retrospective study based on cone beam CT DICOM images of 31 orthognathic surgery patients who had been treated in the University of Glasgow Dental Hospital and School; the patients were randomly selected from the database at the school. Ethical approval to access and use the data was obtained from the West of Scotland Research Ethics Service (Reference 12/WS/0133). The pre-operative CBCT scans were acquired within one month of surgery and the post-operative scans were obtained at a minimum 6 months after surgery using the same CBCT machine (i-CAT Classic, Imaging Sciences, Hatfield, UK). The 3D hard tissue and soft tissue models were segmented from the DICOM file using Maxilim (Medicim, Medical Image Computing, Belgium) with an automated pre-defined HU value for each tissue type determined by the software. The 3D models were then exported as surface files (STL format) in preparation for analysis.

### Preoperative and Postoperative Image Registration

#### A. Surface based registration

**Figure 1:**
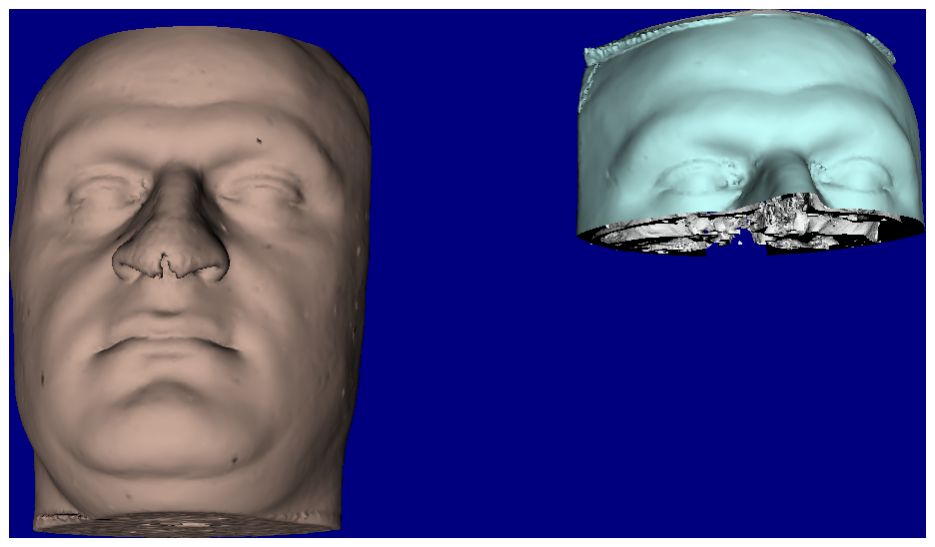
Surface based registration construction of registration template. The forehead (soft tissue) and anterior cranial base (hard tissue) cropped and duplicated from the preoperative model to be used for surface based registration method.

Surface based registration (SBR) of the pre- and post-operative surface images was carried out using VRMesh software (VirtualGrid, Bellevue City, WA). In order to carry out superimposition on a stable structure, an intermediate template common to both models was chosen (Figure 1). This template was a portion of the pre-operative model, which remained unchanged as a result of surgery. For the hard tissue it was the anterior cranial base, which extended to involve the frontal bone, for the soft tissue the forehead and eyes were selected. Superimposition was carried out in two steps: rigid surface registration based on landmarks to bring the two models (preoperative and postoperative) close to each other, for the hard tissue the landmarks identified were the right and left zygomatico-frontal sutures and the centre of fronto-nasal suture. For the soft tissue they were the right and left exocanthi and glabella. This step was followed by ICP (Iterative closest point) registration. These two stages were carried out for both the soft and hard tissue models. The 4 models (2 superimposed pairs) were saved in their new 3D positions in STL format.

#### B. Voxel based registration

Voxel based registration (VBR) was carried out using a specially developed plug-in for Maxilim software. For each patient the pre- and post-operative DICOM images were imported and soft tissue and hard tissue models were segmented as previously described. This enabled better visualisation of the volume of interest which would be chosen for registration. The stable volume of interest included the anterior cranial base and the forehead region. The voxel based registration algorithm was performed based on maximizing mutual information with an iterative translation and rotation of the DICOM image volume to find the best match of the grey scale intensity between the two overlapping DICOM images voxel by voxel [16].

**Figure 2:**
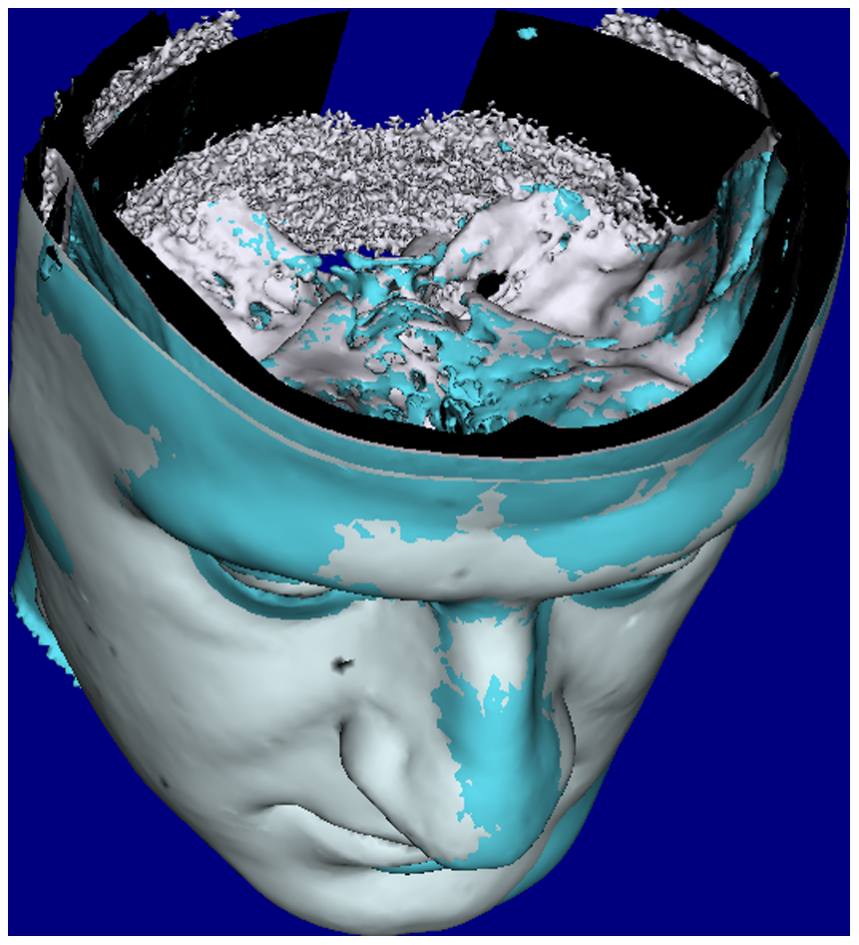
Voxel based registration. The DICOM image including both soft and hard tissues was translated and rotated to the closest fit with its correspondent image.

At the end of the process, the whole postoperative DICOM image stack was registered to the preoperative image position by translating and rotating it as a single unit into a common 3D coordinate system (Figure 2). Upon completion of the registration process the soft and hard tissue models of the preoperative and postoperative DICOM images (4 models in total) were exported and saved in the STL format.

### Data Analysis

**Figure 3:**
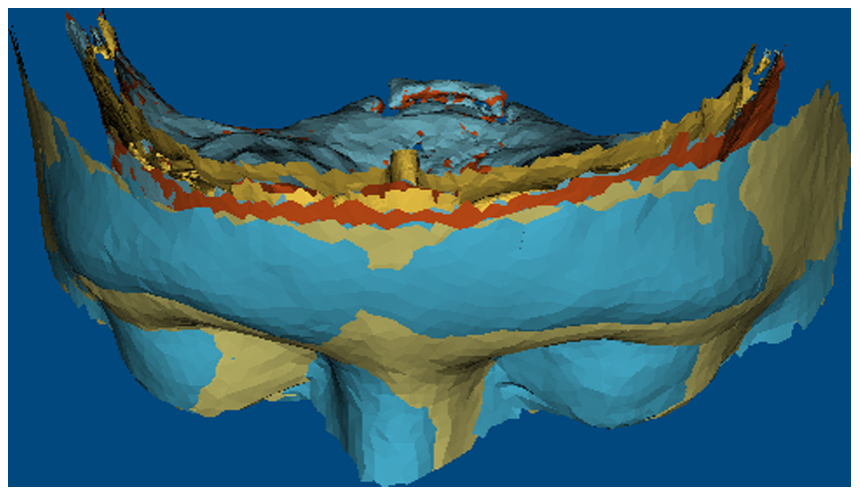
Isolation of the reign of interest. The anterior cranial base (hard tissue models) and the forehead (soft tissue model) were cropped using three planes, two horizontal planes; one above glabella point and the other passing through external canthi. The vertical plane is a coronal plane passing through sella point.

The eight individual STL models were imported into VRmesh software. A standardised view for analyses was chosen for all the image pairs (Figure 3). The inferior limits were determined by a horizontal plane passing through right and left outer canthi; the superior limit was marked by a horizontal plane parallel to the inferior limit and located 20 mm above glabella; the posterior limit was marked by a vertical plane passing through Sella.

The region isolated by these planes in each of the models was exported as a VRML file for final analysis. In this process, the regions of interest in all of the models were cropped to the same dimensions to standardise the region of analysis.

### Analysis of Registration Accuracy

**Figure 4:**
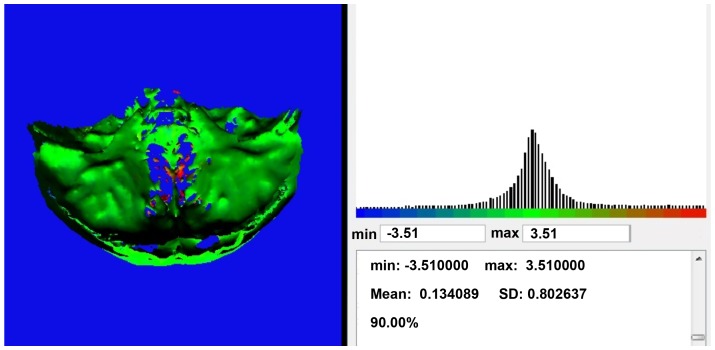
Mean measurements using in-house software. The results of the inter mesh distance measurements includes mean distance, standard deviation and percentage of vertices involved in the test in addition to the diagram showing the distribution of the distances around the mean.

The pre- and post-operative SBR hard tissue models were imported into in-house software developed at the University of Glasgow. The software measured the Euclidian distance of each vertex of the post-operative surface model to the surface of the preoperative model. To exclude any outliers 90% of the points (90th percentile) were used and the mean absolute distance, standard deviation, maximum and minimum distances recorded (Figure 4). This was repeated for SBR and VBR of the hard and soft tissue separately.

### Statistical Analysis

A paired Student *t*-test was used to detect any statistical differences between SBR and VBR for pre- and post-operative images for both soft and hard tissues selected regions (p<0.05). An ANOVA and post-hoc Duncan test was used to detect any significant difference between each method of superimposition and tissue type i.e. hard or soft tissue. A Pearson correlation coefficient was applied to the four superimposition groups (SBR hard, SBR soft, VBR hard and VBR soft) to test the correlation among superimposition groups. A one sample t-test was used to test if the absolute mean difference between the post-operative soft tissue 3D models aligned by each registration method was greater than 0.5 mm.

## Results

**Figure 5:**
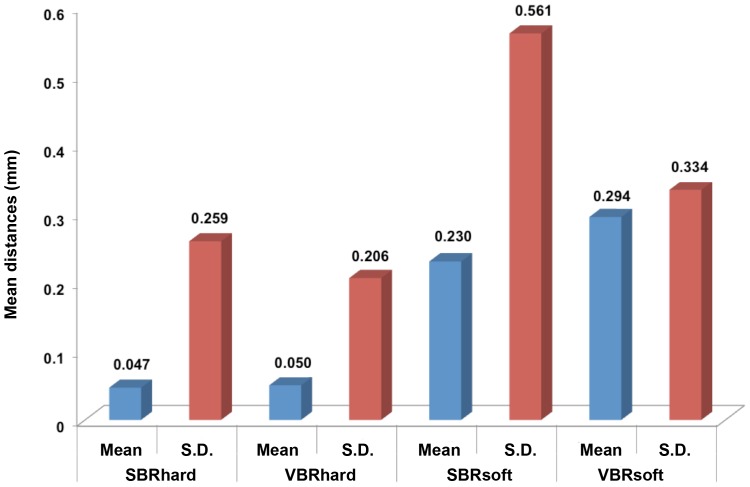
Descriptive analyses for comparing mean distances. The lowest mean distance was recorded for SBR hard while the highest mean distance was recorded for VBR soft. Note that the standard deviation was higher for SBR than VBR in all cases.

Figure 5 shows the descriptive analysis of the four superimpositions groups. The four superimpositions were ranked from the lowest to the highest absolute mean distances between corresponding 3D meshes. Voxel based registration and surface based registration of the hard tissues showed the same values in the absolute mean distances between the models, 0.05 (±0.21) mm and 0.47 (±0.26) mm respectively. For soft tissue superimposition the absolute mean distances between the meshes was larger on the voxel based registration than that on surface based registration, 0.29 (±0.33) mm and 0.23 (±0.56) mm respectively. For both hard and soft tissue the paired Students *t*-test showed no statistically significant difference between the two superimposition methods, (Table 1). Using the mean of the absolute distances between two surfaces as a method of assessment has previously been reported and is an acceptable parameter [17].

**Table 1:** Paired sample t-test to compare means.

	95% CI Lower (mm)	95% CI Upper (mm)	Mean (mm)	SD (mm)	Mean SE (mm)	p-value
SBRhard - VBRhard	−0.01	−0.06	0.00	0.02	0.00	0.392
SBRsoft - VBRsoft	−0.18	0.05	0.28	0.05	0.54	0.243

95% CI = 95% confidence interval.

A one way ANOVA and post hoc Duncan test were used to investigate the statistical significance of the differences between any pair of the four groups (SBR hard, SBR soft, VBR hard and VBR soft). The result of ANOVA test showed a statistically significant difference between the four groups. The post hoc Duncan test showed that the type of tissue i.e. hard or soft tissue influenced the accuracy of superimposition using either surface based or voxel based registration methods. A statistically significant difference was found between superimposition of the soft and hard tissue models within the same method. The difference between the VBR hard and VBR soft superimpositions was statistically significant (p<0.001); the absolute mean difference was 0.23 mm, Table 2. However, the difference between SBR hard and SBR soft was not statistically significant (p = 0.712).

**Table 2:** Paired sample t-test showing the significance of the difference within the groups.

	95% CI Lower (mm)	95% CI Upper (mm)	Mean (mm)	SD (mm)	Mean SE (mm)	p-value
SBRhard - SBRsoft	−0.42	0.29	−0.06	0.96	0.17	0.712
VBRhard - VBRsoft	−0.03	−0.16	−0.23	0.21	0.04	0.000

95% CI = 95% confidence interval.

Statistical correlation between different groups was analysed using a Pearson correlation test, Table 3. VBR hard and SBR hard superimpositions showed a strong positive correlation (r = 0.886). VBR soft and SBR soft showed a weak positive correlation (r = 0.126). This implies that the superimposition of the hard tissue did not show variability between the two methods whereas the soft tissue superimposition showed high variability.

**Table 3:** Pearson correlation coefficient analyses.

		SBRhard	VBRhard	SBRsoft	VBRsoft
SBRhard	Pearson Correlation	1	0.886**	0.190	0.102
	p-value		0.000	0.343	0.613
VBRhard	Pearson Correlation	0.886**	1	0.126	0.182
	p-value	0.000		0.532	0.363
SBRsoft	Pearson Correlation	0.190	0.126	1	0.126
	p-value	0.343	0.532		0.532
VBRsoft	Pearson Correlation	0.102	0.182	0.126	1
	p-value	0.613	0.363	0.532	

**Correlation is significant at 0.01 level (2-tailed).

The one sample t-test showed the absolute mean difference between the pre- and post-operative soft tissue position when VBR was used to align soft tissue images, or SBR was used. The difference was not statistical greater than 0.5 mm (p = 0.73). The clinical significance was determined to be 0.5 mm from a previous study [19].

## Discussion

This study aimed to evaluate the accuracy of voxel based registration compared to surface based registration methodology and to determine if the difference between them is statistically significant. Accordingly, the research method was based on 31 pairs of pre-operative and post-operative CBCT scans of patients treated by orthognathic surgery. The study investigated the accuracy of both methods in registering the post-operative image to the corresponding pre-operative images.

Despite the fact that both methods of registration use the information provided by a CBCT generated DICOM image, voxel based registration deals with the raw information of the DICOM image by comparing the grey scale intensity of the voxels composing the corresponding DICOM images; on the other hand, surface based registration requires an extra step involving 3D model rendering to generate a three dimensional surface mesh model, on which the surface based registration is performed. This additional step may introduce a possible source of error since the algorithm used for segmenting the 3D model depends on the Hounsfield value (HU value) of DICOM images of the CBCT. The form and dimension of the 3D surface model is dependent on the HU value [20] which in turn may be affected by image quality and tissue density. In addition, this extra step increases processing time and implies the need for multiple software packages for 3D model rendering which is unnecessary in the case of voxel based registration.

Another parameter worth considering when comparing the two methods is the amount of information utilised for the purpose of registration. Surface based registration uses the 3D information provided by surface mesh topography of the 3D model, whereas voxel based registration uses the grey scale values of all the voxels imbedded in and around the anatomical structure and is not dependent upon surface features. In other words, surface based registration deals with the “shell” covering the 3D structure while the voxel based registration deals with all the contents of the volume selected, which may theoretically increase the accuracy of the method. However the use of such information implies the need for more efficient computers and a longer processing time [16]. Another consideration is the cost implication and availability of the computer software. Surface based registration utilises a surface mesh, which is routine for conventional computer aided design, and computer aided manufacture (CAD/CAM) software making the cost relatively low and software readily available. On the other hand DICOM registration software is specialised and limited mainly to the medical arena and therefore is more expensive.

Despite the fact that both methods use the ICP algorithm for superimposition, which involves repetitive translation-rotation movement and measurements between the two 3D objects to reach the best matching superposition, the two approaches are considerably different. Surface based registration apply an estimation of the optimal translation and rotation between the three dimensional shapes by minimizing the mean square distance between the surfaces The distance is measured between a specified percentage of the points randomly selected on one 3D mesh and the corresponding 3D surface mesh. However, with voxel based registration the estimation of the optimal translation and rotation between the 3D volumes is determined by the mean square difference in the grey scale intensity between a specified percentage of voxels randomly selected on one image volume and the overlapped voxels in the corresponding one.

Loss of the sharpness of a 3D image during capture may be a source of error due to confusion in the estimation of the grey scale level of the voxels and therefore registration. However, the degree of DICOM image sharpness has a similar effect on the surface based registration but indirectly and may not be detected due to the automatic surface smoothing of the image. The accuracy of 3D model segmentation from a DICOM image is affected by the quality of the DICOM image. In other words, the algorithm will have to decide where to place the boundaries of the hard tissue when building a skull model from a DICOM image with loss of sharpness and the resultant 3D model will represent the estimated dimensions rather than the original.

Four of the samples used in this study were considered as outliers with values reaching up to six times the general attitude of the sample and introducing errors by significantly changing the mean values of all of the superimposition groups. They were excluded from the study sample for this reason.

In all cases, surface based registration demonstrated a higher variability in superimposition as indicated by the larger standard deviation, Figure 5. This may be due to the SBR algorithm relying on well-defined surface features for registration which are present on the hard tissue but are not a prominent feature of the relatively homogenous surface of the soft tissue forehead. With respect to VBR registration, the distribution of the voxel’s grey scale intensity was thought to be the reason for a lower variation in the superimposition process, which was reflected as a lower standard deviation.

Further investigation, using the Pearson correlation coefficient test, was carried out to observe the correlation between different registration methods within each pre- and post-operative data set. A strong positive correlation (r = 0.886) was found between the hard VBR and SBR of the hard tissue models There were weak positive correlations among all other groups of the study. This result highlighted two important observations; firstly, surface based registration for hard tissue was as accurate and consistent as the voxel based registration. A possible explanation may be the high level of feature specific information available on the hard tissue surface which improves the performance of surface based registration. The relatively smooth surface of the soft tissue model reduces the accuracy of the registration and increases the variability of the results. Alternatively, voxel based registration relies on the grey scale intensity of the DICOM image voxels rather than the soft and hard tissue model surface topography, which makes it more consistent in both regions.

The other finding was the weak positive correlation between the soft and hard tissue models registration using voxel based registration (r = 0.126). Unlike surface based registration, the voxel based registration algorithm translates and rotates all the tissues captured in the DICOM image simultaneously. Hence, a strong correlation would be expected between the soft and hard tissue models alignment measurements. This result may be explained by the effect of variation of facial expression during the pre-operative and post-operative image capture and the possibility of soft tissue thickness change as a result of weight changes in the time interval between the two scans. The fact that the voxel based registration algorithm relies on the grey scale intensity of the entire image may result in excluding these small differences in soft tissue contour as outliers during the registration process. This finding suggests that voxel based registration produces a more accurate representation of soft tissue changes as a result of surgery as compared to surface based registration. Surface based registration of the soft tissue aligns the pre and post-operative images irrespective of the underlying hard tissue and therefore will “force” the two surfaces as close as possible, whilst VBR may be restrained by the underlying hard tissue since it is involved in the registration process. The differences between the two methods of registration are unlikely to have any clinical significance [20].

### Conclusions

No statistically significant differences were detected between the voxel based and surface based registration methods. However, voxel based registration showed more consistency in representation of the actual soft and hard tissue positions as indicated by lower mean standard deviation. Soft tissue surface based registration does not take into account changes in tissue thickness.
